# CENP-A, a protein required for chromosome segregation in mitosis, declines with age in islet but not exocrine cells

**DOI:** 10.18632/aging.100220

**Published:** 2010-10-29

**Authors:** Seung-Hee Lee, Pamela Itkin-Ansari, Fred Levine

**Affiliations:** ^1^ Sanford Children's Health Research Center, Sanford-Burnham Medical Research Institute, La Jolla, CA 92037, USA; ^2^ Department of Pediatrics, University of California San Diego, La Jolla, CA 92093, USA; ^3^ Neurosciences, Aging, and Stem Cell Center, Sanford-Burnham Medical Research Institute, La Jolla, CA 92037, USA

**Keywords:** β-cell, replication, pancreas, diabetes

## Abstract

Beta-cell replication dramatically declines with age. Here, we report that the level of CENP-A, a protein required for cell division, declines precipitously with age in an islet-specific manner. CENP-A is essentially undetectable after age 29 in humans. However, exocrine cells retain CENP-A expression. The decline in islet-cell CENP-A expression is more striking in humans than in mice, where CENP-A expression continues to be detectable at low levels even in elderly mice. The mechanism by which CENP-A declines appears to be post-transcriptional, as there was no correlation between CENP-A mRNA levels and age or islet purity. This finding has implications for efforts to induce beta-cell replication as a treatment for diabetes.

## INTRODUCTION

Inducing beta-cell replication as a means of increasing beta-cell mass is a major goal of diabetes research. There has been tremendous controversy about the extent to which beta-cells replicate, but it is becoming increasingly clear that in adult animals and humans, the rate of beta-cell turnover, whether by replication or neogenesis, is low under most circumstances [1,2,3]. In mice, beta-cells have been found to replicate in a small number of physiologically relevant settings, including during embryogenesis and early in postnatal life, during pregnancy, and in response to obesity [4]. In humans, it appears that postnatal beta-cell replication is more variable than in mice, but there are indications that most takes place during infancy [5]. Beta-cell replication declines precipitously with age in mice [1,6] and in humans, where age-related loss of replicative ability and expression of important beta-cell transcription factors occurs [7]. Recently, examination of the rate of lipofuscin in human beta-cells revealed that there was little to no beta-cell turnover in adult humans [2]. A similar finding, also in humans came from examination by radiocarbon dating and BrdU incorporation [3].

While beta-cell replication appears to decline with age, that does not mean that it cannot occur with an appropriate stimulus. In vivo, growth factors such as exendin-4, EGF, and gastrin have been studied as potential inducers of beta-cell replication [8]. In vitro, Hayek and coworkers reported that human beta-cells can be stimulated to enter the cell cycle in vitro when cultured on a complex extracellular matrix [9], but this has been disputed as possibly being due to replication of exocrine cells contaminating the islet preparations [10]. Recently, it was reported that overexpression of cdk6, an important cell cycle regulatory protein, induced human beta-cells to enter the cell cycle [11]. However, there was no evidence of actual proliferation as determined by an increase in the number of beta-cells.

In the absence of a reproducible and efficient means of inducing beta-cells to replicate in vivo or in vitro, studying beta-cell replication must rely on the examination of the expression of proteins that are important in that process [12]. Centromere Protein A (CENP-A) is a 17 kDa member of the histone family. It is over 60% identical in amino acid sequence to histone H3 at the C-terminus, but is highly variable at the N-terminus. It is found in the nucleosomes of active centromeres, where it is found in place of histone H3. Its presence is required for centromere function, with absence leading to chromosome mis-segregation and cell death [13]. It may also play a role in repair of double stranded DNA breaks [14]. CENP-A expression is regulated throughout the cell cycle at both the transcriptional and post-transcriptional levels [15]. However, it is thought to be expressed ubiquitously. To date, there are no studies that have demonstrated age-related changes in the expression of CENP-A.

## RESULTS

### CENP-A protein decreases with age in human islets, but not exocrine cells

**Figure 1.**
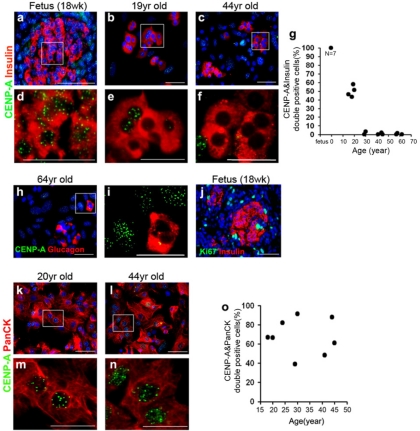
CENP-A protein expression declines with age in human islet cells, but not in exocrine cells. Representative low power (**a-c**) and corresponding high power (**d-f**) images from human pancreases and islet preparations immunostained for CENP-A (green) and insulin (red) in fetal pancreas (**a, d**, N=7), cultured islets from donors of 15-20 years of age (**b, e**, N=4) and cultured islets from donors of 29-62 years of age (**c, f**, N=8). Quantitative analysis of CENP-A and insulin double positive cells as a function of donor age (fetal pancreas were of 18-21 week gestation, N=7)(Linear Regression, R^2^=0.77) (**g**). More than 100 insulin positive cells were scored from each donor. Cells were scored as positive for CENP-A if any immunofluorescence signal was detected. CENP-A expression in alpha-cells (CENP-A-green, glucagon-red) (**h, i**). Human fetal pancreas immunostained for Ki67 (green) and insulin (red)(**j**). CENP-A expression in exocrine cells (CENP-A-green, PanCK-red, N=8)(**k-n**). Quantitative analysis of CENP-A and PanCK double positive cells as a function of donor age (Linear Regression, R^2^=0.0021)(**o**). More than 1000 PanCK-positive cells were scored from each donor. Blue nuclear counterstain was DAPI. Scale bars=50uM.

In the human pancreas, CENP-A protein expression exhibited an inverse relationship with age. In the human fetal pancreas, where 7 different pancreases were examined, 100% of beta-cells exhibited nuclear staining for CENP-A (Figure 1a, d). This staining occurred in a punctuate pattern and no difference was observed in the pattern of CENP-A between beta-cells and other cells in the pancreas. CENP-A expression in beta-cells declined rapidly with age and by age 30 was undetectable (Figure 1b, c, e, f, quantitated in g). Alpha-cells also lost CENP-A with age (Figure 1h, i). In contrast to beta-cells, exocrine cells continued to express CENP-A at approximately the same level from ages 18-45 (Figure 1k-n, quantitated in o).

### The decline in CENP-A protein in islets is less dramatic in mouse islets than in human islets

**Figure 2.**
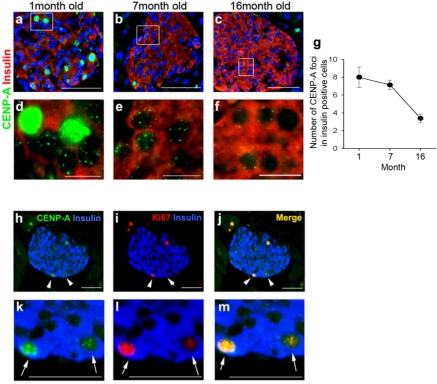
CENP-A expression in mouse beta-cells as a function of age. Representative low power (**a-c**) and corresponding high power (**d-f**) images from mouse pancreases of different ages immunostained for CENP-A (green) and insulin (red). Quantitative analysis of CENP-A and insulin double positive cells as a function of age (1 month old mice, N=4; 7 month old mice, N=4; 16 month old mice, N=3). Data are mean ± SEM (**g**). The number of foci of CENP-A staining per beta-cells was quantitated. More than 20 islets were counted from each mouse. Low power (**h-j**) and corresponding high power (**k-m**) images from mouse pancreases immunostained for CENP-A (green), insulin (blue) and Ki67 (red). Arrowheads (high power views) and corresponding arrows (low power views) indicate dividing beta-cells (positive for insulin and Ki67) that also exhibit a high level of CENP-A (green). Blue nuclear counterstain is DAPI. Scale bars, a-c=50uM, d-f=10uM, h-m=50uM.

In the mouse, CENP-A protein expression also declined with age (Figure 2a-f, quantitated in g). However, unlike the human pancreas, it continued to be detectable in the majority of beta-cells even at 16 months, the last time point examined. While CENP-A continued to be expressed in most beta-cells, the number of CENP-A foci in the nucleus declined with age (Figure 2d, e, f, quantitated in g). In contrast with the human pancreas, mouse CENP-A was upregulated in dividing cells compared with non-dividing cells (Figure 2h-m). In the human fetal pancreas, the only place where a substantial number of dividing cells could be detected in the pancreas, there were no cells expressing high levels of CENP-A, despite the presence of dividing cells expressing Ki67 (Figure 1a, j).

### CENP-A mRNA levels do not change with age

**Figure 3.**
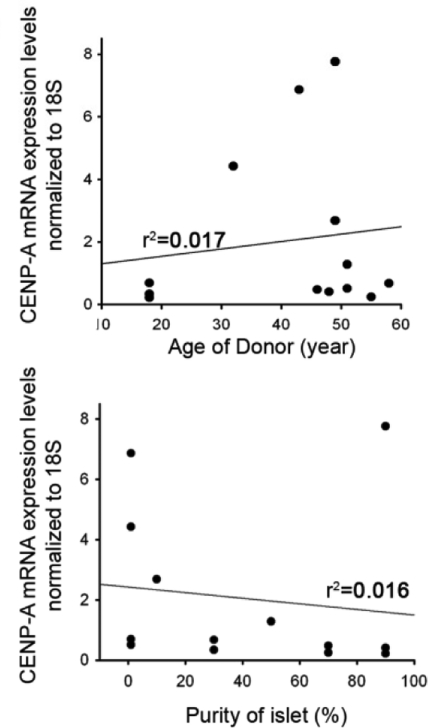
Human CENP-A mRNA expression is indepen-dent of donor age in islet and exocrine cells. Isolated endocrine and exocrine cells were analyzed for CENP-A mRNA levels with quantitative RT-PCR and normalized to 18S mRNA (donors of 18-58 years of age, N=13).

To determine whether the decline in CENP-A expression was transcriptional or post-transcriptional, we measured CENP-A mRNA levels by quantitative RT-PCR in preparations of cultured human islets. There was no correlation between CENP-A expression and age (Figure 3a, R^2^=0.017). In addition, there was no correlation between the level of CENP-A mRNA and islet purity, which is consistent with the absence of age-related effects on CENP-A mRNA expression, given that we found no change in CENP-A expression in the exocrine pancreas with increasing age (Figure 3b, R^2^=0.016). Thus, the decline in CENP-A expression in islets appears to be at the post-transcriptional level.

## DISCUSSION

While it is clear that beta-cell replication declines with age in humans and mice, the responsible mechanism(s) remain to be elucidated. Prior to this report, the only cell cycle regulatory protein that had been shown to exhibit age-related change was the cyclin dependent kinase inhibitor p16INK4A, which increases in beta-cells with age in vivo [16,17], and in human beta-cells stimulated to divide in vitro [18]. Recently, it was shown that Ezh2, a histone methyltransferase that is part of a Polycomb transcriptional repressor complex, represses p16INK4A in beta-cells and declines with age in both mice and humans, leading to an increase in p16INK4A expression [16]. Interestingly, the kinetics of decline described for Ezh2 appear similar to what we found for CENP-A, with both exhibiting substantial loss by early adulthood. Also of note, the increase in p16INK4A and decrease in CENP-A is beta-cell specific, as in neither case is there a change with age in the exocrine pancreas [17] (Figure 1).

CENP-A has not been recognized to be a senescence marker and so its loss in beta-cells over time might reflect a phenomenon other than simple cellular senescence. CENP-A is regulated by FoxM1 [19] and its mRNA increases following partial pancreatectomy [20]. The ability of CENP-A to access centromeric chromatin is regulated by histone acetylation, and treatment with the histone deacetylase inhibitor trichostatin A allowed CENP-A to bind to centromeres in cells lacking lacking the centromere protein hMis18α [21]. Treatment of human islets with trichostatin A did not result in increased CENP-A expression (unpublished results). However, we find here that the decline in CENP-A is due to post-transcriptional regulation, which has not been previously reported.

Because CENP-A is required for cell division, the finding that it declines with age suggests the possibility that increased p16INK4A is not solely responsible for the decline in beta-cell replication with age, particularly in humans. It cannot be determined whether CENP-A is completely absent from beta-cells or whether its expression has decreased below the limit of detection of immunostaining. Decreasing CENP-A by RNAi, which reduces but does not eliminate gene expression, led to a profound defect in cell division, suggesting that even partial loss of CENP-A could be significant [22].

Clearly, the possibility exists that more defects in aged beta-cells remain to be uncovered. The finding reported here does not rule out a model in which there is a common upstream regulator of CENP-A, p16INK4A, and possibly other proteins that play a role in age-related replicative decline, but the fact that p16INK4A is transcriptionally regulated while the decline in CENP-A appears to be post-transcriptional suggests that multiple mechanisms may be operative. The possibility that multiple events are involved in distinct proliferative defects could greatly complicate efforts to enhance beta-cell replication.

## METHODS

### Cells and Tissues

Human fetal pancreases at 18-21 gestational weeks and cold ischemia time within 12-24 hours were obtained from Advanced Bioscience Resource, Oakland, CA. Tissue procurement was done in accordance with Institutional IRB regulations. Murine pancreases were harvested from FVB mice purchased from Harlan Sprague Dawley. Principles of laboratory animal care' (NIH publication no. 85-23, revised 1985; http://grants1.nih.gov/grants/olaw/references/phspol.htm) were followed.

Primary adult human islets and exocrine tissues were obtained from the NIH Islet Cell Resources-Administrative and Bioinformatics Coordinating Center (ICR-ABCC), JDRF, and University of Alberta Islet Isolation Center, Canada. Primary cells were cultured in 5.5mM glucose RPMI /10% FBS/1% Pen/Strep (Invitrogen) on HTB9 matrix plates or collagen plates (Becton Dickinson) in 5% CO2 incubator for 2-3 days to form monolayers prior to fixation and analysis [23].

### Immunohistochemistry

Human fetal pancreas and mice tissues were fixed in cold methanol and embedded in OCT freezing media. Samples were sectioned to a mean thickness of 5 microns. Cultured cells and tissues were fixed with cold methanol for 10min, blocked for 1hr, and incubated with primary antibody for 16hr at 4°C.

Antibodies used were as follows: anti-CENP-A (Abcam ab13939, Cell Signaling 2048S), anti-insulin (Santa Cruz sc-9168, sc-8033), anti-glucagon (Sigma G2654, Abcam ab8055), anti-PanCK (Dako Z0622) and anti-Ki67 (Dako M7249, M7240). For fluorescent imaging, samples were incubated with Alexa 488 (Invitrogen), Rhodamine (Jackson) fluor-labeled anti-mouse/rabbit/rat and nuclear counterstained with DAPI (Sigma).

The antibody to human CENP-A has been used extensively in the past [24,25]. It was further characterized by us by transfection (Lipofectamine 2000, Invitrogen) into mouse 4T1 cells of an expression vector encoding a human CENP-A-GFP fusion gene [14]. GFP-expressing cells stained only with human CENP-A antibody and there was no staining in untransfected mouse cells (Supp. Figure).

For quantitative analysis, cells were scored as positive for CENP-A if any immunofluorescence signal was detected in nucleus. More than 1000 PanCK and 100 insulin positive cells were counted from each human donor. More than 20 islets were analyzed in the mouse pancreas for each animal.

### Quantitative RT-PCR

Total RNA extracted from human islets and exocrine cells with RNeasy Mini kit (Qiagen). 1ug of total RNA prepared for cDNA and 2ul of cDNA used as template. Human CENP-A PCR conditions were 5 min at 94 °C, followed by 28 cycles of 94 °C for 45 sec, 60 °C for 45 sec, and 72 °C for 45 sec. Quantitative RT-PCR used the Opticon Real-Time System (BioRad), with SYBR Green (BioPioneer). RT-PCR for 18S rRNA was used for normalization.

## SUPPLEMENTAL FIGURE

Supplemental Figure. Specificity of human CENP-A antibody. A plasmid expressing a human CENP-A-GFP fusion protein [14] was transfected into 4T1 mouse cells with Lipofectamine and immunostained for human CENP-A antibody (**a-c**, green-GFP, red-human CENP-A, yellow-merge). Human CENP-A antibody specifically stained GFP-expressing cells in mouse 4T1 cells and HeLa cells transfected with control plasmid (**d-f**). Blue nuclear counterstain was DAPI. Scale bars=50uM.
