# Supplementary figures and images for: CENP-A, a protein required for chromosome segregation in mitosis, declines with age in islet but not exocrine cells
Source: Aging (Albany NY). 2010 Oct 29;2(11):785–90. doi: 10.18632/aging.100220 (PMC3006021; doi:10.18632/aging.100220)

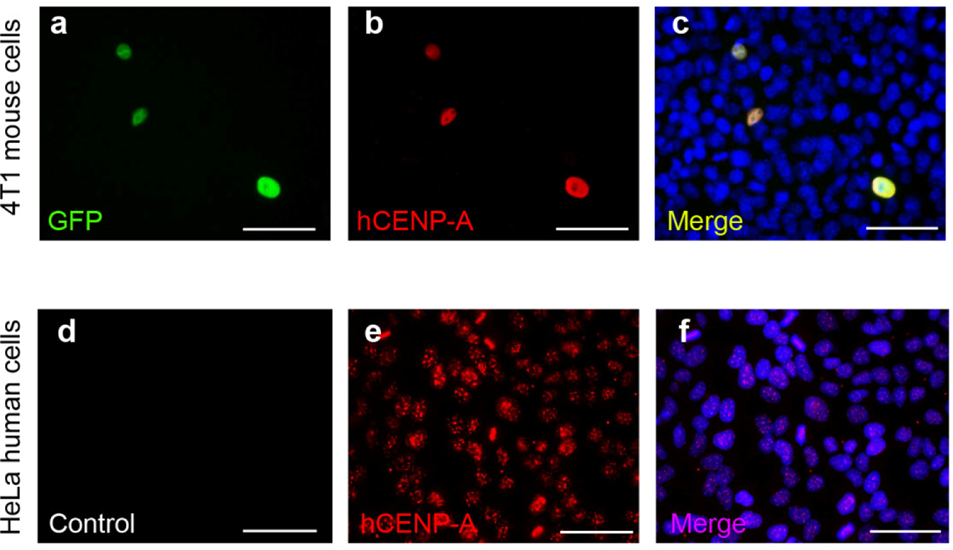

Supplement: Supplemental Figure. — A plasmid expressing a human CENP-A-GFP fusion protein [14] was transfected into 4T1 mouse cells with Lipofectamine and immunostained for human CENP-A antibody (a-c, green-GFP, red-human CENP-A, yellow-merge). Human CENP-A antibody specifically stained GFP-expressing cells in mouse 4T1 cells and HeLa cells transfected with control plasmid (d-f). Blue nuclear counterstain was DAPI. Scale bars=50uM. [file aging-02-785-s001.tif]
